# Feasibility and implementation of a personalized, web-based exercise intervention for people with cystic fibrosis for 1 year

**DOI:** 10.1186/s13102-021-00323-y

**Published:** 2021-08-19

**Authors:** Barlo Hillen, Perikles Simon, Sebastian Schlotter, Oliver Nitsche, Viola Bähner, Krystyna Poplawska, Daniel Pfirrmann

**Affiliations:** 1 Department of Sports Medicine, Disease Prevention and Rehabilitation, Johannes Gutenberg University, Mainz, Germany; 2 Medical Department of Pediatrics Pulmonology, Allergology and Cystic Fibrosis, University Medical Centre, Langenbeckstraße 1, 55131 Mainz, Germany

**Keywords:** Personalized telemedicine, Clinical exercise therapy, Chronic diseases, Exercise prescription, Long-term intervention

## Abstract

**Background:**

Regular participation in exercise is important for people with cystic fibrosis (CF). Therefore, we implemented a personalized, web-based exercise intervention over the course of one year for people with CF. The aims were to investigate the feasibility of the intervention and to evaluate changes in exercise participation, lung function, and exercise capacity.

**Methods:**

In total, 11/17 participants [aged 12–52 years; FEV_1_%pred. 72.3 (SD: 17.3)] were included in the final data analysis. Every week, the participants received an individual training recommendation at the start and uploaded their training report on our website at the end of each week. The number of training minutes and sessions performed were analyzed over 13 four-week training sections. The participation in exercise (physical activity questionnaire), lung function and exercise capacity were assessed at baseline (T0), after 12 weeks (T1) and after 52 weeks (T2).

**Results:**

A training duration of 178 min (SD: 75.5) and 3.3 (SD: 0.89) training sessions could be achieved weekly. In the first four-week training section, the participants performed 137.31 (SD: 95.7) minutes of training, with an increase of 42% in the third training section (195.01, SD: 134.99). Minutes of training reported on the questionnaire increased by 39.7% from T0 (179.38 min, SD: 120.9) to T1 (250.63 min, SD: 124.1) but decreased at T2 (166.88, SD: 155.4). There were slight decreases in lung function (FEV_1_ − 3.9%pred.; FVC − 1.9%pred.) and slight increases in exercise capacity (VO_2peak_ + 1.5 ml/min/kg; six-minute-walk-test-distance + 26 m). Noticeably, five participants experienced deteriorations in their FEV_1_ of more than 5% but simultaneously experienced improvements in the parameters of exercise capacity of more than 5% throughout the year.

**Conclusions:**

The web-based concept was feasible for the participants over the course of a year and supported exercise participation. The improvement in exercise capacity due to increased exercise participation over a prolonged period of time, despite a decrease in lung function, should be further investigated. Finally, if integrated into usual care, this approach could facilitate the prescription of regular personalized exercise and promote exercise participation in the daily lives of people with CF.

## Background

Nowadays, it is well known that exercise performed adequately and regularly has physiological [1], psychological [2] and immunological [3] benefits to human health. Most patients with chronic diseases can benefit from appropriate daily exercise [4]. People with cystic fibrosis (CF) can benefit from regular exercise participation not only in terms of increased respiratory capacity but also the prevention of comorbidities, as the current therapies and new medications have markedly prolonged the lifespans of people with CF [5].

During the past decade, many research groups have observed the positive effects of structured exercise on aerobic physical capacity, health-related quality of life and pulmonary function [6–10]. An improvement in lung function [11, 12], and reductions in infection and inflammation [13], the hospitalization rate [14] and fatigue [15] have also been observed due to exercise or physical activity. Ward et al. confirmed that exercise is a fundamental therapeutic intervention for people with CF [10]. Radtke et al. observed no adverse side effects of exercising [8]. Consequently, regular and adequate participation in exercise can be assumed to be an essential part of the lives and management of people with CF.

Nevertheless, evidence regarding the efficacy of exercise training in people with CF is still limited, and the most effective exercise frequency, intensity, type and time still need to be identified [8, 10]. Moreover, there is a lack of evidence regarding effective strategies for promoting participation in physical activity at home that is sustained for at least six month by people with CF [16]. Web-based exercise interventions are believed to be a promising solution [17, 18].

We developed a personalized, web-based exercise program to promote regular participation in exercise. The use of this web-based concept has already been shown to be feasible for patients with CF over a period of eight weeks [18]. The aim of this study was to examine the feasibility of this web-based exercise intervention and its impact on participation in exercise over the course of an entire year. Additionally, we evaluated self-reported participation in exercise, lung function and exercise capacity before, during and after one year of the intervention.

## Methods

### Study design

This observational, web-based exercise intervention lasted 52 weeks and is a part of the cystic fibrosis online mentoring for microbiome, exercise and diet (COMMED) study. Within COMMED a CF physician, a dietician and a sport scientist could personally interact with the participants via a website and directly at three specific time points. The CF physician was present at each testing timepoint. The dietician could provide nutritional recommendations at each testing timepoint. Whereas the information exchange with the medical doctor and the dietician was voluntary, the participants were obligated to exchange information with the sports scientist weekly. The parameters regarding participation in exercise throughout the intervention were analyzed after 52 weeks. Additionally, we compared self-reported physical activity, lung function and exercise capacity at baseline (T0), after the first 12 weeks (T1) and after 52 weeks (T2).

### Participants

The participants were recruited at the medical Department of Pediatric Pulmonology, Allergology and Cystic Fibrosis of the University Medical Centre in Mainz, Germany. People with CF who met the following criteria were included in the study: age > 12 years, FEV_1_%pred. > 28 and < 100 and/or lung clearance index (LCI) > 9. The exclusion criteria were orthopedic, rheumatic, cardiovascular or neurological contraindications; no internet access; and an acute exacerbation. Written informed consent was obtained from all participants (or their parents or guardian). The study was approved by the Human Ethics Committee Rhineland-Palatinate (837.288.16 (10, 607)) and conformed to the Code of Ethics of the World Medical Association (Declaration of Helsinki).

### Design of the web-based exercise program

**Fig. 1:**
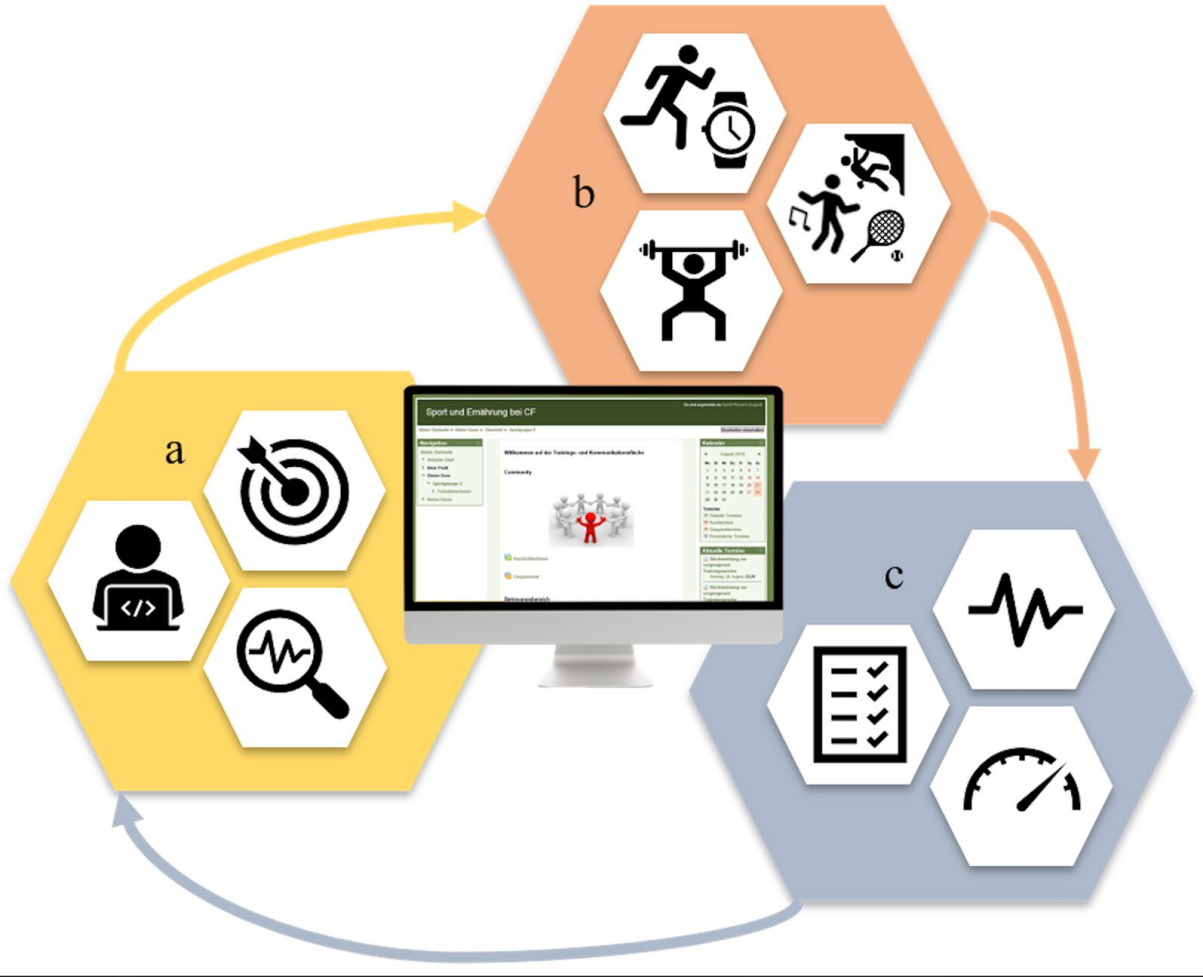
Design and overview of the weekly feedback loop between the participants and the sports scientist. *Legend*. The screen in the middle of the figure shows the design of the website. Part a of the concept illustrates the initial and weekly training analysis and individual training adaptation designed by the sports scientist. Part b represents the combined training options, such as endurance training, strength training and preferred physical activities. The blue hexagon illustrates the third essential part of the program, which was feedback provided by the participants to the sports scientist via an uploaded, hand-written exercise protocol at the end of each week. Based on this protocol, the sports scientist could adapt the training load for the following week

Figure 1 illustrates the design of the weekly feedback loop between the participants and the sports scientist that operated via the website, which is described in the following section.

At baseline, the participants performed an incremental modified walking test (s. Fig. 3) on a treadmill until they reached voluntary maximum exhaustion to identify any exercise-related risks or symptoms, maximum exercise capacity and individual training zones.

Afterwards, the participants were registered on the website and received training material, including three resistance bands (Pinofit®), a heartrate monitor (Polar FT7), a Redondo ball and two tennis balls. On the website, the participants could download all exercise descriptions, illustrated manuals or videos; upload their training protocols; receive their weekly training recommendations; and chat with other participants.

At the start of every week, the participants received a message on the website containing a tailored training plan for the following week. This training plan comprised endurance, strength, flexibility and coordination training sessions and integrated preferred physical activities.

Continuous and interval training were recommended for endurance training. The participants could choose their preferred endurance exercise type (brisk walking, running, cycling, rowing, swimming). The recommended exercise intensity was mainly based on the individual anaerobic threshold (IAT) and the variation in oxygen saturation (SpO_2_) measured during the exercise test.

Strength (module 1), coordination (module 2) and flexibility (module 3) training sessions were created by the sports scientist and uploaded with descriptions, pictures and videos on the website. Module 1 to 3 could be performed with the training material that had been provided or with everyday objects in the home environment. For instance, the disease-related strength training module consisted of five complete training sessions: (1) “training with own body weight”; (2) “training with free weights or filled water bottles”, (3) “training with resistance bands”, (4) “training with the Redondo ball”, (5) “training on the gymnastic mat”. Sets and repetitions of each session and the specific combination of the three modules could be varied and adapted by the sports scientist.

As a third additional option, the participants could pursue their preferred physical activities, such as horseback riding, tennis or climbing.

At the end of each week, the participants had to upload their hand-written exercise protocol (JPEG/PDF) to the website. This exercise protocol contained the performed exercise sessions, the duration of each session and the individual load and discomfort in each session. The individual training load was indicated by the mean heart rate per session (HR), measured by the HR monitor, and the rated perceived exertion (RPE 0–10). Rated perceived discomfort (RPD 0–10) was the indicator for exercise tolerance.

**Fig. 2:**
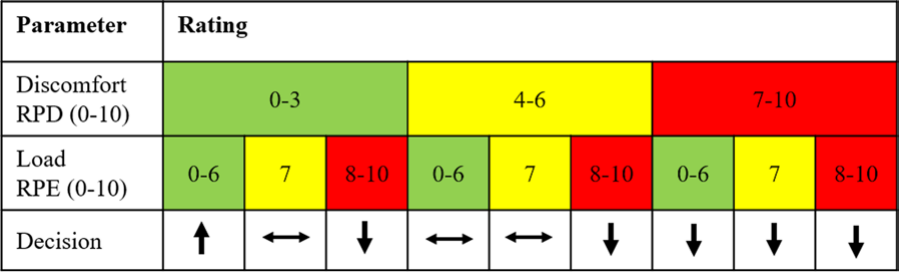
The decision strategy used to adapt the training load. Legend. After each training session, the participants rated their training session according to the perceived discomfort (0–10) and perceived exertion (0–10). RPD was divided into low (0–3), moderate (4–6) and high (7–10). RPE was also divided into three categories: very light to moderate (0–6), vigorous (7), and hard to maximum effort (8–10). Arrows in the last line illustrate the training load adaptation for each of the nine possible parameter combinations. This decision strategy originated from and the figure is adapted from [18]

After the participants provided feedback via the weekly uploaded training protocol, the sports scientist adapted the exercise program for the upcoming week. Figure 2 shows the decision strategy employed by the sports scientist to adapt the training for the following week. During periods of acute infection or exacerbation or while on holiday, participants could interrupt the training program.

### Definition and assessment of exercise participation as primary outcome

The primary outcome was participation in exercise over the course of one year. The exercise participation parameters were the time spent training and training frequency over 52 weeks for the entire group and for each individual. Therefore, 52 hand-written training protocols for each participant (n = 572) were evaluated. Table 1 presents the exercise participation-related parameters.

**Table 1:** Evaluated parameters of exercise participation

Nr.	Exercise participation parameter
1.1	Individual training time/week (mean_min)
1.2	Individual training sessions/week (mean_sessions)
1.3	Individual training weeks (n/52; %)
2.1	Group training time/training section^a^ (mean_min)
2.2	Group training sessions/training section (mean_sessions)
3.1	Domain 4 IPAQ—leisure (moderate, vigorous): training minutes at T0, T1, T2
3.2	Domain 4 IPAQ—leisure (moderate, vigorous): training sessions at T0, T1, T2

^a^Section: The 52 weeks of the intervention were divided into 13 4-week training sections

Additionally, the participants completed the “Physical Activity Questionnaire” (IPAQ) [19], on which they reported their physical activity during the past seven days preceding the intervention (T0), after 12 weeks of the intervention (T1) and after 52 weeks of the intervention (T2). The number of minutes spent training and frequency reported in domain four of the IPAQ, “leisure” (vigorous; moderate), were evaluated.

### Assessment of lung function and exercise capacity as secondary outcomes

Lung function (FEV_1_, FVC (%pred.)) and maximal and submaximal exercise capacity (VO_2peak_, six-minute walk test distance (6MWTD (m)), maximum work rate (Power_peak_ (w)), work rate at the individual anaerobic threshold (Power_IAT_ (w)), and heart rate at the individual anaerobic threshold (HR_IAT_ (beats/min)) were the secondary outcomes. These parameters were measured at baseline (T0), after 12 weeks (T1) and after 52 weeks (T2) of the intervention.

Forced expiratory volume in one sec (FEV_1_) and forced vital capacity (FVC) (%pred.) were assessed using flow-volume spirometry (MasterScope®Body, CareFusion, Germany) in accordance with the criteria of the American Thorax Society/European Respiratory Society. FEV_1_ and FVC were determined as the highest of three acceptable measurements and expressed as a percentage of the predicted reference value according to the Global Lung Function Initiative [20].

The 6-min walk test (6MWT) was conducted to assess submaximal exercise capacity.

The 6MWTD was the output of the 6MWT. The participants had to achieve their maximum distance within 6 min while moving at their fastest possible walking speed.

**Fig. 3:**
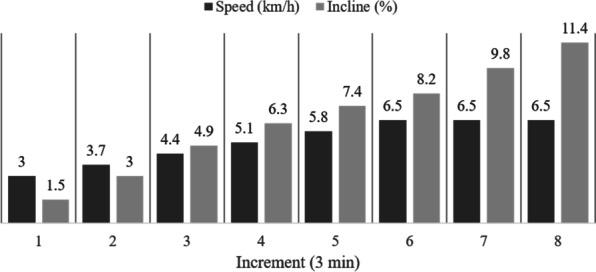
Modified walking test protocol performed on a treadmill during cardiopulmonary exercise testing

The cardiopulmonary exercise test (CPET), including a lactate analysis, was performed to assess submaximal and maximal exercise capacity. A modified incremental walking test protocol [21] was performed on a treadmill (Saturn, HP Cosmos®) with increasing speed (km/h) and incline (%) until maximum exhaustion was reached (s. Fig. 3). The increments lasted three minutes.

Ventilation of O_2_ (VO_2_) and CO_2_ (VCO_2_) (Ergostick, Geratherm®), lactate (L) (EKF diagnostics®; Winlactat 3.0 Mesics GmbH), heart rate (HR) (ECGpro, AmedTec®), peripheral capillary oxygen saturation (SpO2) and rate of perceived exertion (RPE) [22] were measured during the 6MWT and the CPET. L samples were taken from the ear lobe after each increment of the CPET. The individual anaerobic threshold (IAT) was determined according to the Dickhuth model [23]. VO_2peak_ was determined to be highest VO_2_ during the last 30 s of the CPET.

### Statistical analysis

Microsoft Excel (2013) and IBM SPSS Statistics version 23.0 were utilized. We analyzed the exercise participation data exploratively (mean, standard deviation, median, min, max, range, 95% confidence intervals). We performed repeated-measures ANOVA for the analysis of the primary and secondary outcomes. To test the violation of the assumption of a normal distribution, the Shapiro Wilk-test was conducted. Mauchly’s W-test was performed to test for sphericity. The Greenhouse–Geisser correction (ε) was applied when the assumption of sphericity was not met. The significance level of *p* < 0.05 was set for all statistical analyses.

## Results

Initially, 17 participants were included in this study. Six of those participants dropped out, and 11 participants were included in the final data analysis for the entire year. This participant group had a mean age of 32.9 years (SD ± 11.4), mean FEV1%pred. of 72.3 (SD ± 17.3) and a mean BMI of 22.7 kg/m^2^ (SD ± 2.18) (Table 2). Of the 11 included subjects, six were males and five were females. The dropout rate of 35.3% occurred for the following reasons: no response after three weeks (n = 1), difficulties with changing life circumstances (n = 1), severe pulmonary exacerbation (n = 1), infections immediately before T2 (n = 2) and acute kidney failure (n = 1).

### Exercise participation throughout the year

**Fig. 4:**
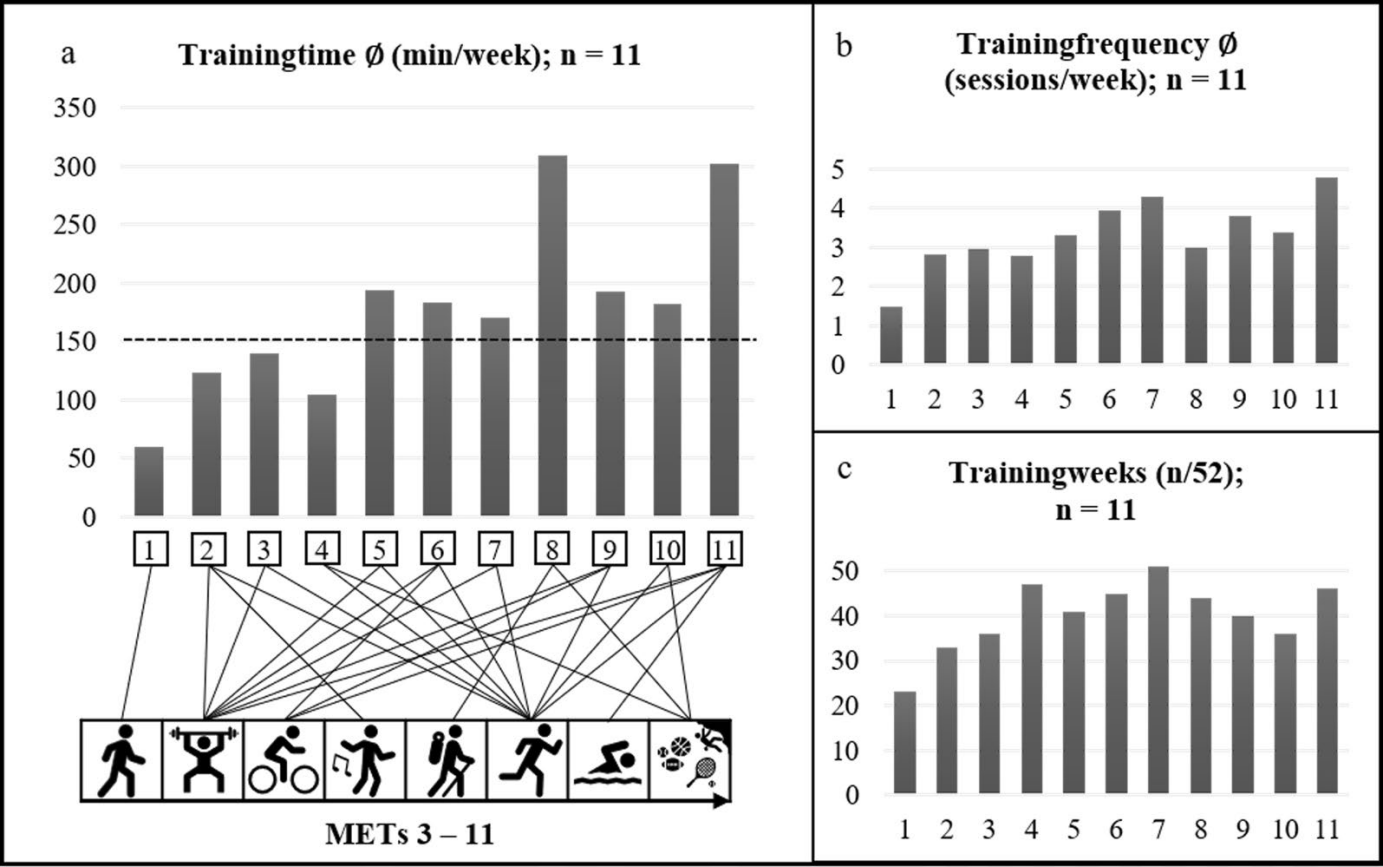
Exercise participation of each participant over the course of the 52 weeks of the intervention. Legend. **a** illustrates training time (min/week) in combination with performed exercise types and METs for each participant. METs, metabolic equivalents (1 MET = 3.5 male; = 3.15 VO_2_ (ml/min/kg)). METs for each exercise type are in accordance with [24]. **b** Illustrates the mean performed training sessions per week during the intervention. **c** Shows the active training weeks from the intended 52 week of intervention

The weekly exchange between the sports scientist and each participant remained feasible throughout the year. Communication was interrupted briefly during vacations and in some cases due to inpatient stays. The number of training sessions and the training duration in minutes were extracted from 572 handwritten training protocols. Table 2 and Fig. 4 show the descriptive analysis of the groups’ exercise participation during the 52 weeks of the intervention.

**Table 2:** Overview of the parameters of exercise participation

(N = 11)	Training weeks/52; %^a^(SD)	Training time/week^b^	Training sessions/week^c^
Mean	40.2 (7.49); 77.3% (14.8)	178 (75.5)	3.32 (0.886)
Median	41; 79%	182	3.32
Range	28; 53%	249	3.30
Min	23; 45%	59.9	1.48
Max	51; 98%	309	4.78

^a^Number and percent of training weeks participated in among the 52 weeks of the intervention

^b^Training time (min) per active training week within the intervention

^c^Training sessions per week within the active training weeks

Subjects 8 and 11 achieved the most exercise training time (> 300 min/week), most training frequency (> 4 sessions/week) and participated in 85% [8] and 88% [11] of the predetermined training weeks. To achieve this high level of exercise participation, subject 8 chose hiking and climbing as the main activities, whereas subject 11 preferred swimming, running, cycling, resistance exercise and horseback riding. Therefore, subject 8 had a higher overall exercise intensity than subject 11 had.

**Fig. 5:**
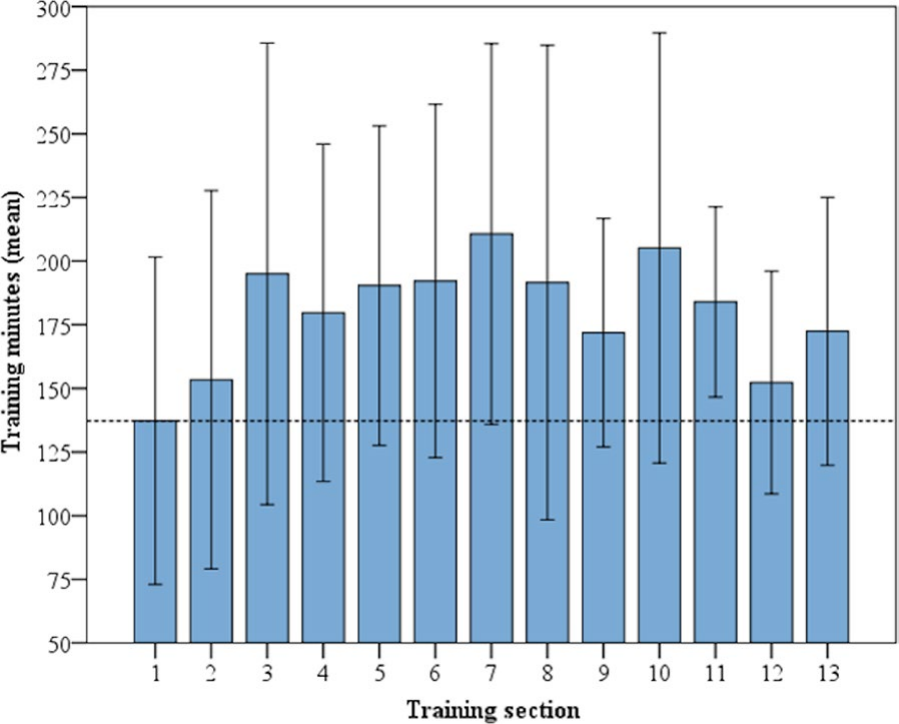
Mean training minutes per four-week training section (95% confidence intervals)

For the analysis of the variation in exercise participation throughout the year, the 52 weeks were divided into 13 four-week training sections. In the first training section, the participants participated in 137.31 (SD: 95.7) training minutes. In all of the following training sections, the number of minutes spent training were. The highest mean numbers of training minutes were achieved in the third (195.01, SD: 134.99), seventh (210.7, SD: 104.56) and tenth (205.18, SD: 125.72) training sections. From the first four-week (1–4) section to the third four-week section (9–12), the number of training minutes increased by 42%. Figure 5 shows the mean training minutes achieved for each training section.

**Fig. 6:**
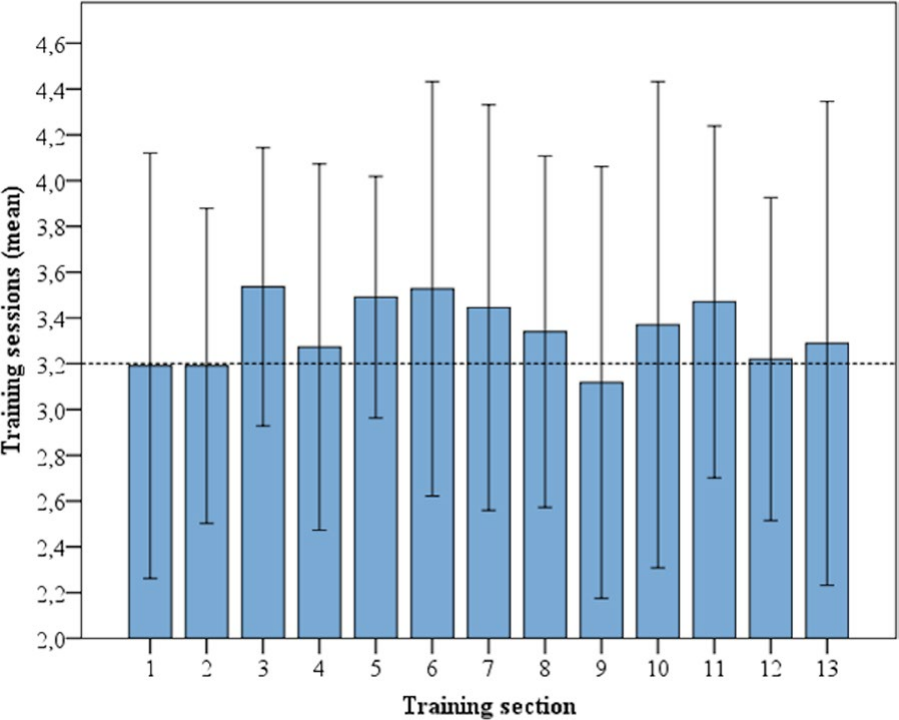
Mean training sessions per four-week training section (95% confidence intervals)

In the first training section, the participants participated in 3.2 (SD: 1.4) training sessions. Only in section nine was the number of mean weekly training sessions lower. The maximum numbers of training sessions occurred in training sections three (3.5, SD: 0.9), six (3.5, SD: 1.3) and seven (3.4, SD: 1.3). Figure 6 shows the training sessions completed in each training section.

The mean numbers of minutes spent training and training sessions in each for the 13 four-week training sections for the seven participants could be included in the repeated-measures ANOVA due to the listwise exclusion of missing data. Missing data resulted from training sections for which no mean value could be calculated for a participant, because in at least one of the four weeks the training was interrupted. The data were normally distributed at all timepoints (*p* > 0.05) except in training section 2 (*p* = 0.01) for the number of minutes spent training. Sphericity was not observed (*p* = 0.000) between the training sections. Greenhous Geisser correction was applied. Repeated-measures ANOVA revealed no significant difference between the training sections with regard to training minutes (*p* = 0.366) or training sessions (*p* = 0.478). The distinct range of the confidence intervals, i.e., the heterogeneous participation in training in the group, led to the non-significant differences between each training section.

### Self-reported physical activity at T0, T1, T2

The self-reported physical activity of eight participants who completed the IPAQ at T0, T1 and T2, could be included in the repeated measures ANOVA due to the listwise exclusion of missing data. Missing data resulted from the absence of information provided in the questionnaire at least at one timepoint (3/33). The data were normally distributed at each timepoint (*p* > 0.05), and sphericity was observed (*p* = 0.806). These 8 participants reported a mean of 179.38 min (SD: 120.9) at T0, 250.63 min (SD: 124.1) at T1, and 166.88 (SD: 155.4) at T2. There was no significant difference between the timepoints (*p* = 0.120). The self-reported number of minutes spent training increased by 39,7% from T0 to T1. This identified increase was no longer evident after 52 weeks.

### Lung function and exercise capacity at T0, T1, T2

From T0 to T2, there were slight decreases in the mean FEV_1_ −3.9%pred. (*p* = 0.102) and in the FVC of −1,9%pred. (*p* = 0.575); slight increases in the VO_2peak_ of + 1.5 ml/min/kg (*p* = 0.413), the 6MWTD of + 26 m (*p* = 0.287) and the Power_peak_ of + 0.11 w/kg (*p* = 0.362); and slight decrease in the Power_IAT_ of −0.01 (*p* = 0.714) and the HR_IAT_ of −4 b/min (*p* = 0.230) (Table 3).

**Table 3:** Comparison of exercise capacity and lung function between timepoints

	TP	FEV^a^	FVC	VO_peak_^b^	6MWTD	Power_peak_	Power_IAT_	HR_IAT_
Mean (SD)	T0	72.3 (17.3)	87.3 (13.4)	28.5 (6.5)	590 (87.1)^a^	1.51 (0.5)	1.13 (0.3)	148 (13.3)
	T1	71.0 (19.8)	85.6 (13.7)	29.4 (7.0)	601 (62.3)	1.56 (0.5)	1.14 (0.4)	147 (11.6)
	T2	68.4 (20.8)	85.4 (15.6)	30.0 (9.0)	616 (56.3)	1.62 (0.6)	1.12 (0.3)	144 (10.9)
rmANOVA (p)		0.102	0.575	0.413	0.287^b^	0.362^b^	0.714^b^	0.230

TP, time point; T0, baseline assessment; T1, assessment after 12 weeks; T2, assessment after 52 weeks; FEV_1_, forced expiratory volume in one second (%predicted); FVC, forced vital capacity (%predicted); VO_2_peak, oxygen uptake at maximum work rate (ml/min/kg); 6MWTD, six-minute walk test-distance (m); Power_peak_, maximum work rate (w/kg); Power_IAT_, work rate at individual anaerobic threshold; HR_IAT_ = heartrate (beats/min); SD, standard deviation; rmANOVA, repeated-measures analysis of variance

^a^Data violated the assumption of a normal distribution

^b^Greenhouse-Geisser correction was applied

**Fig. 7:**
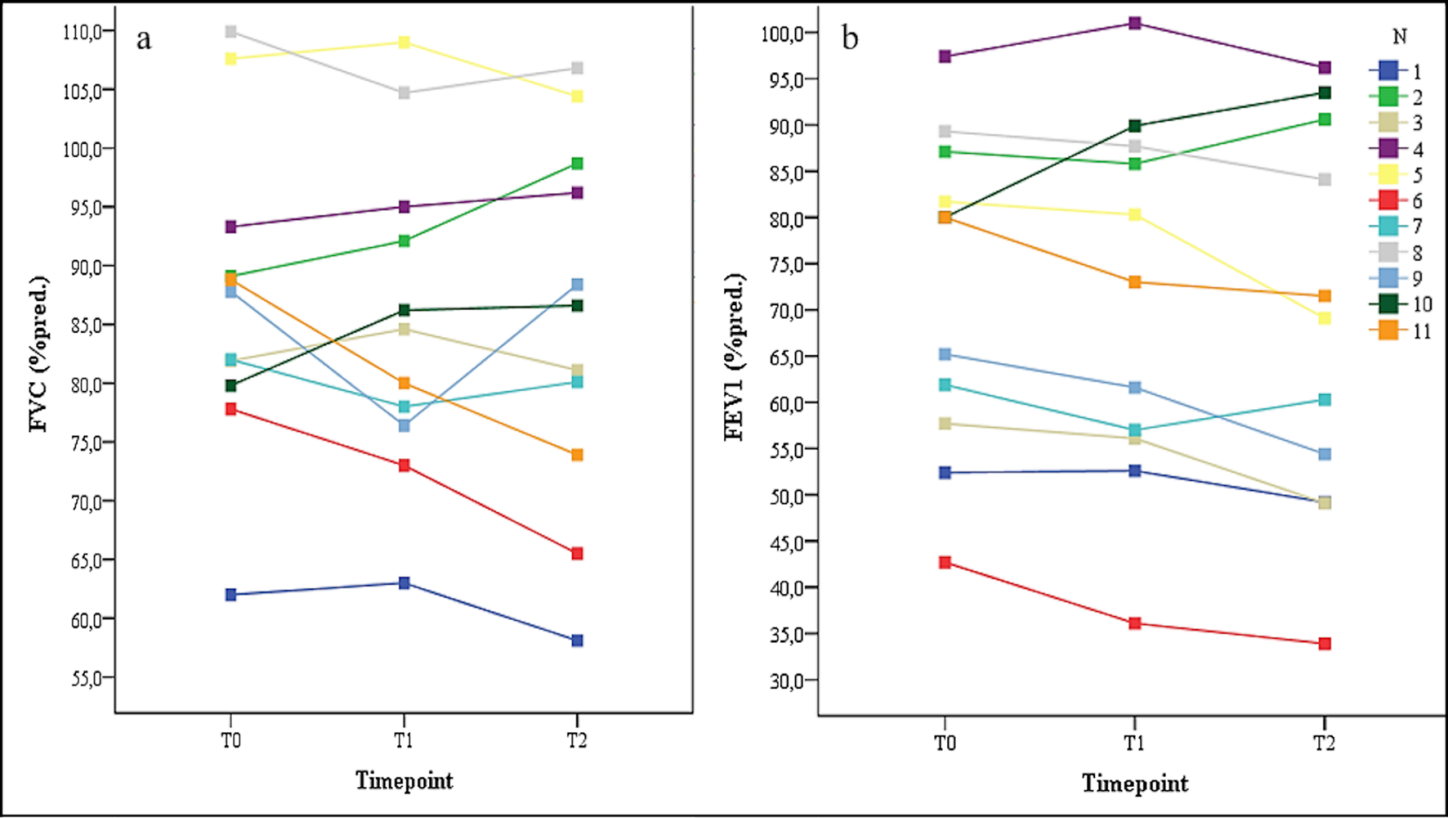
Changes in VO_2peak_ and 6MWTD from T0 to T1 to T2 for each participant. Legend. T0, baseline assessment; T1, assessment after 12 weeks; T2, assessment after 52 weeks; VO_2peak_ = oxygen ventilation at maximum work rate (ml/min/kg); 6MWTD = six-minute walk test distance (m)

**Fig. 8:**
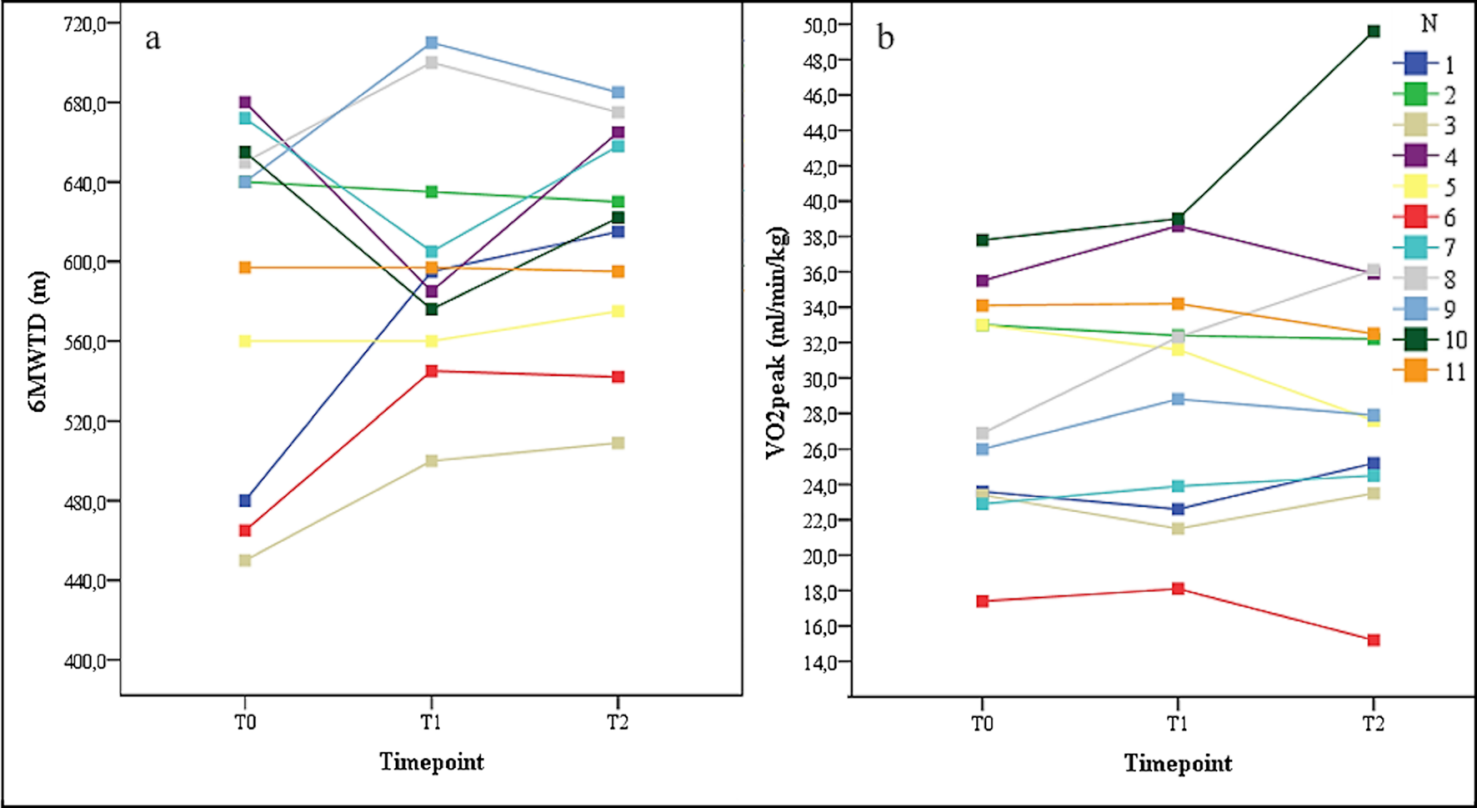
Changes in FEV_1,_ and FVC from T0 to T1 to T2 for each participant. Legend. T0, baseline assessment; T1, assessment after 12 weeks; T2, assessment after 52 weeks; FEV_1_ = forced expiratory volume in one second (percent predicted); FVC = forced vital capacity (percent predicted)

There were heterogeneous changes in the secondary outcomes in the interindividual comparison. The changes in exercise capacity are shown in Fig. 7. Figure 8 shows the changes in lung function for each participant across all tests. Seven participants increased their 6MWTD, seven increased their VO_2peak_, nine decreased their FEV_1_ and seven decreased their FVC.

Under the exploratory assumption that a five percent change in pulmonary function and exercise capacity from T0 to T2 could be considered clinically relevant, the following observations can be made: Subjects 1, 3, 6, and 9 improved their 6MWTDs by more than 5%. Only participant 10 experienced a worsening of the 6MWTD by slightly more than 5%. Participants 1, 7, 8, 9, and 10 improved their VO_2_peak by more than 5%. Participants 5 and 6 experienced decreases in their VO_2_peak of more than 5%. Only participant 10 improved the FEV_1_ by more than 5%. Participants 1, 3, 5, 6, 8, 9, and 11 experienced reductions in their FEV_1_ of more than 5%. Participants 1, 2, 3, 4, 5, and 10 improved their FVC by more than 5%. No participant experienced a reduction in the FVC. Considering the individual changes in secondary outcomes as a whole, it is interesting that participants 1, 3, 6, 8, and 9 experienced reductions in their FEV_1_ by more than 5% but experienced improvements in the parameters of exercise capacity of more than 5%.

## Discussion

Taking into account that according to the WHO guidelines for physical activity, 150 min/week is recommended for healthy adults [25] and that overall adherence of young people to exercise recommendations is generally poor, the overall achieved mean training time of 178 min per week is noteworthy. The mean training time was lower in the first four-week section (< 150 min/week) than in the following 12 training sections (> 150 min/week). This means that a sustained increase in training minutes can be assumed to be indicative of the long-term suitability impact of the intervention.

However, a progressive increasing trend in exercise participation could be observed only until the seventh four-week training section. In the following training sections, exercise participation fluctuated. These fluctuations could be related to the normal health changes in people with CF that occur over the course of a year or to reduced reporting. The exercise participation protocol had to be written by hand and then uploaded to the website as a scan or image. This appeared to be more a result of the circumstances of the study than a necessary element of the program, and the automatic acquisition of training data, and the transformation of the web-based concept into a mobile application could solve this issue in the future. Moreover, fluctuations in exercise participation could also be due to the time of year, as infections are more common in colder months and engagement in physical outdoor activities usually decreases somewhat during those months as well. Additional indoor training opportunities at home such as endurance training on an ergometer might support exercise participation during the colder periods of the year.

As shown in the results, the self-reported number of minutes spent training and sessions in domain 4 of the IPAQ were also higher at T1 than before the intervention, which strengthens the assumption that the exercise intervention could support exercise participation in people with CF. Notably, the exercise participation assessed at T0 and T1 on the IPAQ was higher than the reported exercise participation reported in our protocols. However, the percentage increase in exercise participation from T0 to T1 (IPAQ) or from the first training section to the third training section (exercise protocol) was similar. The potential overreporting on the IPAQ has also been reported previously [26]. An automated, objective measurement of exercise participation utilizing reliable wearables might further verify this observation in future studies. At T2, the exercise participation reported on the IPAQ was lower than that at T1, which might attenuate the long-term suitability of the intervention.

Considering these observations, the long-term feasibility of the web-based concept is clear, and exercise participation did increase, especially during the first three to seven month of the intervention; however, the long-term suitability (> 7 month) should be further investigated.

Remarkably, despite slight increases in VO_2peak_ and 6MWTD, slight decreases in FEV_1_ and FVC occurred after our intervention. Even a “stabilization” of these parameters over the course of a year in people with progressive disease [27] would be considered a positive outcome. Most interestingly, five participants experienced reductions in their lung function but enhancements or no changes in their exercise capacity. However, we cannot assume that this “stabilization” occurred only due to our intervention. One reason is the lack of a control group and potentially influential factors, such as medication, which come into play during a one-year intervention under real-world conditions. A recent study, which evaluated a large international sample of people with CF, confirmed the importance of exercise capacity as a predictor for long-term mortality [28]. In this context, even a small increase in exercise capacity, despite an observed reduction in lung function in our study, may have a positive effect on long-term survival in our participants. However, while we have now shown the practicability of a long-term web-based approach, the concrete health effects need to be determined.

There are other reports of telehealth approaches to the management of CF [28–33]. Cox et al. [29] demonstrated the feasibility and acceptability of the internet-based program “Active Online”, which lasted eight weeks, in combination with phone calls every second week in ten adults with CF (mean age 30 years and mean FEV_1_ 50%pred.). They suggested conducting a randomized controlled trial to examine the effect on participation in physical activity. In 2018, Chen et al. [30] addressed the issue of the risk of cross-infection in the population of CF patients. Therefore, they examined the use of a live-streaming platform that offered supervised and interactive group exercises. In this examination, ten patients had to perform 30 min of aerobic resistance training three times per week for six weeks. The patients completed 85% of the training sessions, and they reported enjoying the training and gave the platform a high rating. The effects on physiological parameters were not reported. The effectiveness of a home-based video game program for young CF patients was tested by Del Corral et al. [31]. The research group observed that six weeks of the video game intervention during rehabilitation improved the participants’ six-minute walk test distance and health-related quality of life, among other parameters. The patients completed a 30- to 60-min training session five days per week, but they included very young patients (mean 13 years old) with a mean FEV_1_ of 82.7% predicted. A year later, Tomlinson et al. [34] presented the concept of “video calling via skype”. Nine patients completed eight weeks of this intervention, during which an exercise therapist supervised all sessions. They achieved 68% compliance but also reported no changes in the health parameters. All of these studies were short-term interventions.

Recently, elaborate and promising study protocols for web-based RCTs have been published. Hebestreit et al. [30] published a study protocol for “Activate-CF” involving a “partially supervised conditioning program” based on the use of pedometers, with an international, multicenter randomized controlled trial design. Lang et al. [31] presented a study protocol for a 12-months multimodal telehealth-based outpatient physiotherapy service and compare the usual outpatient physiotherapy service with the telehealth intervention CyFiT, eHAB®. This software allows real-time video sharing, the measurement of motion in various joint, and virtual group-based sessions combined with the use of an activity tracker. Therefore, it remains to be determined how these approaches affect exercise participation in people with CF and influence their lung function and exercise capacity. Innovative person-centered, web-based algorithms aiming in automatically increase and measure physical activity, set personalized physical activity goals, and determine the type and duration of physical activity [35] could also be considered in the design of future studies.

## Limitations

Some limitations of our study must be taken into account. The most important is the small sample size and its heterogeneity due to the inclusion of participants at every stage of CF. This limitation, combined with a lack of a control group, potentially attenuates the power of the reported p-values, which should rather be considered as a supplement to the performed exploratory analysis. A general estimation of the most effective exercise type, duration, intensity and frequency for people with CF cannot be derived from the results. Additionally, over the course of an entire year, our participants needed continuous changes in medications and suffered from acute infections or pulmonary exacerbations, which influenced the outcome. The analysis of exercise participation was based on self-reported protocols. Factors such as social desirability or obliviousness could have caused reporting bias. Nevertheless, self-reporting allows a mentor to receive individual feedback from participants about their current perceptions, efforts and training progression. The recruitment of more participants, the inclusion of a control group, the automation of training data acquisition, the measurement of preintervention exercise participation and the restriction of the exercise options could help overcome the above-mentioned limitations in future studies.

## Conclusions

The web-based concept was feasible for the participants over the course of an entire year and supported their exercise participation. The weekly feedback loop between participants and the sports scientist enabled the provision of regular personalized exercise counselling. Automatic acquisition of the training data and a mobile application could further improve this web-based concept. The potential increase in exercise capacity due to increased participation in exercise over a prolonged period of time, despite a decrease in lung function, should be further investigated. Finally, if integrated into usual care, this approach could facilitate the prescription of regular personalized exercise and promote exercise participation in the daily lives of people with CF.

## Data Availability

The datasets generated and/or analyzed during the current study are not publicly available because they contain sensitive patient data but are available from the corresponding author upon reasonable request.

## Abbreviations

CF: Cystic fibrosis
COMMED: Cystic fibrosis online mentoring for microbiome, exercise and diet
CPET: Cardiopulmonary exercise testing
FEV_1_: Forced expiratory volume in one sec
FVC: Forced vital capacity
HR: Heartrate
HR_IAT_: Heartrate at individual anaerobic threshold
IAT: Individual anaerobic threshold
IPAQ: International physical activity questionnaire
L: Lactate
LCI: Lung clearance index
Power_IAT_: Watt at individual anaerobic threshold
Power_peak_: Watt at maximum exhaustion during cardiopulmonary exercise testing
S_P_O_2_: Peripheral capillary oxygen saturation
RPE: Rate of perceived exertion
RPD: Rate of perceived discomfort
T0: Baseline
T1: After 12 weeks of the intervention
T2: After 52 weeks of the intervention
VCO_2_: Ventilation of CO_2_
VO_2_: Ventilation of O_2_
VO_2peak_: Highest VO_2_ during the last 30 s of the CPET
